# The use of Introduction, Situation, Background, Assessment, and Recommendation handover in the COVID-19 pandemic and non-COVID clinical settings: a systematic review and meta-analysis

**DOI:** 10.3389/frhs.2025.1380948

**Published:** 2025-08-07

**Authors:** Carlo Lazzari, Marco Rabottini

**Affiliations:** Department of Health Sciences, International Centre for Healthcare and Medical Education, Bristol, United Kingdom

**Keywords:** handover, handoff, ISBAR, SBAR, COVID-19, outcome, systematic review, meta-analysis

## Abstract

**Introduction:**

The Introduction, Situation, Background, Assessment, and Recommendation (ISBAR) approach to clinical handovers assists healthcare providers in sharing information about patients within clinical teams and across health sectors while reducing information gaps and medical errors. However, despite its significance, uncertainties remain about the clear outcomes of applying ISBAR and training, especially in settings managing COVID-19 and those not dealing with the pandemic.

**Methods:**

This review was conducted following the PRISMA guidelines. All the selected articles underwent a progressive check for bias and validity using GRADEpro GDT and RoB-2 as per Cochrane guidelines. This review utilized a meta-analysis of 29 studies and a critical narrative review of seminal articles to condense the non-numerical findings. All articles were checked for heterogeneity with the *I*^2^ coefficient. The extracted effect size was the common language effect size (CLES), with 95% confidence intervals.

**Results:**

ISBAR strengthens communication skills in clinical teams, increases self-confidence and efficacy among healthcare practitioners, improves interprofessional communication, reduces medical errors, and enhances patient safety.

**Conclusions:**

Our systematic review confirms that ISBAR handover improved the quality of care during the COVID-19 pandemic and non-COVID clinical practice. The limitation of this study is related to the lack of randomized controlled trials and blinding. Almost all studies were cross-sectional, which only provides information regarding associations but not causation.

## Introduction

Coronavirus disease (COVID-19), caused by the SARS-CoV-2 virus, typically results in moderate to severe respiratory illness in most people infected with this virus, who recover without requiring special care. However, certain individuals may develop a severe infection and require medical attention. Older adults and those with underlying health conditions—such as cancer, diabetes, cardiovascular disease, or chronic respiratory disorders—are more likely to experience severe illness when infected. Additionally, COVID-19 can infect people of any age (1). The COVID-19 cases in the UK have followed the global pandemic's trend—there have been 20,629,892 cases since the pandemic was declared in the UK in 2020, with 5,911 admissions in a week and 981,596 people hospitalized as of 27 February 2023 (2). The pandemic also impacted all surgical and medical specialties. Hospitalization and intensive care are required for moderate to severe cases, including non-invasive and invasive ventilation, antipyretics, antivirals, antibiotics, and steroids (3). During a COVID-19 emergency, the clinical teams working with the patient must coordinate their handovers to develop a unified strategy for a pathology affecting multiple organs (3).

Clinical handovers (CHs) involve transferring, whether temporarily or permanently, professional accountability and responsibility for part or all of a patient's or group of patients' care to another individual, family member, legal guardian, or professional organization through verbal, written, or electronic exchanges of patient information and duty among healthcare providers (HCPs) (4, 5). CHs include verbal, face-to-face, written, email, video, or electronic notes on a patient's current clinical conditions, treatment plans, and any assessment and clinical results produced by the professionals developing the handovers (4, 5). A handover involves transferring accountability from the sender to the receiver to reduce confusion about patients' conditions. It clarifies the patients' current state, presentation, diagnosis, and treatment and explains the necessary care management in a specific context and setting through verbal or electronic communication (6). During handovers, data and information about a patient are shared among caregivers, within a team of caregivers, and between the hospital and the patient's family or the patient himself (5).

In more detail, CHs involve transferring responsibility for assessment and treatment, sharing information about clinical conditions, and reviewing patient cases. This process enables continuity of care, shared care plans, and the transfer of duties for patient care and assessment to another team member or different teams to whom the handover is directed or who have equal responsibility for the patient (7–9). In hospitals, handovers usually happen after a shift (in the UK, there are about three handovers each day on average) (7) Thus, CHs (or “clinical handoffs” in the USA) are the temporary (e.g., daily clinical notes, patient progress, and liaison assessment) or final (e.g., discharge from hospital and referral to another team or structure) transfer of information and responsibility for care to another professional team member or group from a different setting, hospital, or community (10). CHs are conducted in various ways during routine daily practice: (a) during verbal handovers, healthcare professionals discuss patients; (b) occasionally, nurses talk about patients while reading notes from family members about the patient's presentation, assessment, or clinical conditions; (c) sometimes, handovers occur at the patient's bedside so that the patient's current presentation, symptoms, and conditions can help fill out the necessary information during CHs (10).

Information refers to any data communicated between people via a shared system of representations, symbols, or behavior (11). The main information communicated during handovers is clinical data about patients. Despite the importance of structured communication through CHs, between 25% and 40% of adverse patient care events, 27% of instances of medical misconduct, and over 70% of warning clinical occurrences are caused by breakdowns in verbal and written CHs regarding patients and among healthcare personnel (12). A review of about 23,000 medical malpractice cases found that over 7,000 cases—nearly 2,000 avoidable deaths and 80% of serious medical errors—were connected to caregiver miscommunication during patient handovers (13). Based on research analyzing 16,165 electronic records in Michigan, interprofessional communication has notable flaws, such as missing essential messages, omitted communication goals, distorted physical or temporal message contexts, absent key participants, and unclear or lost information (14).

Interprofessional communication can be either synchronous or asynchronous; synchronous channels include meetings, ward rounds, handoffs, and spontaneous conversations (15). Asynchronous communication and CHs may also happen through prescription orders, written progress reports, computerized patient notes, and whiteboards (15). The four or five steps of patient CHs form the basis for the situation, background, assessment, and suggestion (SBAR) approach and its variations, which include Introduction, Situation, Background, Assessment, Recommendation, and Suggestion (ISBARS) and SBAR plus Kindness (K-SBAR) (16–19):

• *An introduction* briefly describes the individual or professional providing the evidence; healthcare practitioners (HCPs) who write the handover notes disclose their identity, role, and motivation for creating the document. It also includes biographical data about the patients, such as their name, age, gender, social circumstances, housing, address, ethnic background, and other personal details, such as how the patient came into contact with the healthcare provider providing the handover.
• *Situation* details the patient's condition and summarizes any current issues the team needs to know about; the healthcare providers share and communicate the patient's relevant background information regarding his or her current situation.
• *Background* summarizes the patient's medical and psychiatric history; the HCPs list the significant events that the interprofessional team believes have contributed to the current issue.
• *Assessment* is the clinical evaluation of a patient; healthcare providers share their perspective on the issues, describe what they believe is happening, complete their specialized assessment, and suggest one or more potential solutions.
• *Recommendation* emphasizes what the patient's future care plan should include; the HCPs propose a course of action to address the issue under review and within their area of expertise.

The theoretical framework of this study is based on the Donabedian model, which divides care into three distinct areas: the structure in which care is provided; the process through which interactions between patients and caregivers take place; and the impact of care on the health status of the patient or population, referred to as outcomes (20, 21). Structure or input measures evaluate the attributes of the service provided, such as promoting data sharing during handovers and ensuring their completeness (20, 21). Process measures are the parts of the service operation that influence the intended outcome, such as training healthcare providers to ensure proper transfer of patient communication (20, 21). The impact of the service on patient health outcomes and the extent to which the desired outcomes have been achieved are reflected in outcome measures, such as a reduction in the number of incidents involving missing patient data during handovers (20, 21).

The primary aim of this systematic review is to comprehensively identify and synthesize salient literature examining the implementation of the Identify, Situation, Background, Assessment, Recommendation (ISBAR) handover protocol within diverse healthcare contexts. This review aims to compare and critically assess the reported outcomes associated with ISBAR adoption, focusing on both clinical effectiveness and the implications for patients’ biopsychological and relational well-being. Specifically, the review evaluates ISBAR's role in enhancing communication accuracy, reducing adverse events, fostering interprofessional collaboration, and improving patient engagement and safety. By integrating quantitative and qualitative findings, the study aims to generate an evidence-informed understanding of how ISBAR contributes to systemic improvements in healthcare delivery and nurtures compassionate, patient-centered care. The review also explores contextual factors influencing ISBAR's efficacy, such as training modalities, setting-specific adaptations, and cultural determinants, with a view to guiding future implementation and policy development.

## Methods

### Review questions

1. *What are the key characteristics and documented outcomes of the SBAR/ISBAR handover framework in both COVID-19 and non-COVID clinical settings?* This question aims to identify the structural components, contextual adaptations, and measurable effects of the SBAR/ISBAR protocol across various healthcare environments. Emphasis is on comparing its operational effectiveness in high-stress settings—such as pandemic response units, emergency departments, and ICUs—with routine care settings. The review seeks to evaluate how ISBAR impacts clinical handover accuracy, decision-making processes, interdisciplinary communication, and patient safety. Special attention is paid to differences in outcome reporting, including decreases in adverse events, improvements in workflow continuity, and enhancements in biopsychosocial aspects of patient care during both pandemic and non-pandemic conditions.
2. *What are the perceived barriers and enabling factors for implementing the SBAR/ISBAR handover framework in diverse healthcare systems?* This question explores the practical and contextual factors that influence the adoption and ongoing use of SBAR/ISBAR. It considers organizational, cultural, and interpersonal challenges such as resistance to change, differences in training effectiveness, hierarchical dynamics, and digital interoperability issues. Facilitators examined include institutional support, integration into professional education, standardization efforts, and leadership backing. The aim is to highlight the complex conditions that either hinder or promote ISBAR's implementation—helping to develop strategies that are culturally adaptable, ethically sound, and resilient during systemic disruptions like public health emergencies.

### Inclusion criteria

We selected studies focusing on handovers based on the SBAR/ISBAR framework that are applicable across all clinical settings. The systematic review (SR) included qualitative, quantitative, and mixed-method studies from peer-reviewed journals. We also included policy papers and other reviews on the topic. Additionally, we only used sources published in English or accessible in English. We included policy papers and national guidelines from UK professional regulatory organizations. Studies presenting meta-analyses were excluded. We also considered results from handover training. To broaden our research, we included quantitative, qualitative, and policy papers, as well as policy articles on SBAR/ISBAR in mental and medical settings to expand the review's scope. We used the population, intervention, comparison, outcomes, and settings (PICOS) framework for selection analysis.

### Exclusion criteria

We excluded studies on handovers when using the ISBAR framework. We also omitted gray literature, Internet sources, opinion papers, and government reports. Other exclusion criteria included studies where SBAR/ISBAR was only a minor finding and the main focus of the investigation was not on it. Additionally, we excluded other meta-analyses.

### Population of interest

This review focused on a population of healthcare professionals (HCPs) at various levels of clinical practice, training, or education. These included physicians, nurses, pharmacists, surgeons, psychiatrists, and allied health professionals such as radiographers and occupational therapists. Eligible individuals were either actively employed in healthcare settings or involved in formal educational programs, ranging from undergraduate coursework to postgraduate clinical training and ongoing professional development.

To be included, studies needed to examine the application or evaluative outcomes of SBAR or ISBAR handover protocols within clinical or educational healthcare settings. Studies were required to involve one or more of the aforementioned professional groups and report empirical findings—whether derived from quantitative, qualitative, or mixed-methods designs—pertaining to communication processes, implementation outcomes, patient safety, or clinical efficacy. Only articles published in peer-reviewed journals and available in full-text English were considered. Relevance to both COVID-19 and non-COVID clinical contexts was also a prerequisite to ensure broader applicability across healthcare conditions.

Conversely, studies were excluded if they involved non-healthcare populations, such as administrative personnel or patient cohorts lacking relevant clinical or communication outcome data. Works that did not explicitly address SBAR/ISBAR frameworks or only mentioned them peripherally without analysis were excluded. Studies lacking outcome data related to handover quality, patient impact, or operational implementation were also omitted. Editorials, opinion pieces, conference abstracts, and gray literature that were not peer-reviewed were considered ineligible, as were publications in languages other than English without available translations.

### Interventions

This review included studies that examined the application, training, or knowledge development related to the SBAR/ISBAR communication framework within healthcare settings. Interventions were eligible if conducted in clinical or educational environments under both COVID-19 and non-COVID-19 circumstances. Studies were considered relevant if they involved healthcare professionals or trainees from health services, acute and community care, or other clinical specialties. Eligible research encompassed structured communication programs, simulation exercises, curriculum integration, and protocol use in real-world practice, as long as they provided data on feasibility, impact, or knowledge outcomes.

Studies were excluded if they did not focus on SBAR/ISBAR frameworks as a central intervention or if they lacked empirical evaluation of training, implementation, or communication-related outcomes. Research conducted outside healthcare contexts or involving non-clinical populations was considered irrelevant. Interventions that did not relate to clinical handover or interdisciplinary exchange or those that merely mentioned SBAR/ISBAR without a methodological focus were excluded. Studies not available in English or not peer-reviewed, including opinion pieces, editorials, and conference abstracts, were omitted.

### Comparisons

This review included studies that used comparative methods to evaluate the effectiveness, acceptability, and clinical impact of SBAR/ISBAR communication frameworks. Eligible comparisons involved assessing participants' performance before and after receiving structured SBAR/ISBAR training, focusing on changes in handover accuracy, confidence, and communication clarity. Studies were also included if they compared respondent groups based on experience, professional role, or engagement with handover protocols—especially regarding ISBAR usage fidelity and quality. Additional criteria extended to research comparing participant satisfaction before and after implementing clinical handover systems (CHs), as well as comparing clinical outcomes related to SBAR/ISBAR implementation in both COVID-19 and non-COVID-19 healthcare settings.

Studies were excluded if they lacked a clear comparative component or did not report outcome-based contrasts attributable to SBAR/ISBAR interventions. Research that did not involve pre-/post-intervention designs, intergroup comparisons, or assessment of clinical handover efficacy was considered ineligible. Additionally, studies that reported only descriptive or anecdotal accounts without analytical comparison—such as narrative summaries or editorial commentaries—were excluded. Comparative studies conducted in non-healthcare domains or involving tools unrelated to SBAR/ISBAR were also omitted.

### Outcomes

Studies were included if they assessed the impact of the ISBAR communication framework on healthcare-related outcomes. Specifically, eligible studies examined ISBAR's effect on nurses' clinical skills and the quality of care provided. Research focusing on carers' confidence, self-efficacy, and readiness to engage in effective handover communication was also included. Studies reporting on interprofessional collaboration and communication improvements among healthcare teams using ISBAR, especially within multidisciplinary settings, were considered relevant. Additionally, investigations evaluating ISBAR's influence on patient safety—including the reduction of adverse events, improvements in handover continuity, and enhanced risk identification—were included, as long as outcomes were empirically measured.

Studies were excluded if they did not evaluate the outcomes of ISBAR implementation or training related to healthcare delivery or professional development. Research lacking empirical data on nursing performance, caregivers' confidence, interprofessional communication, or patient safety was deemed ineligible. Studies that mentioned ISBAR without assessing its impact, as well as those limited to conceptual discussions without reporting outcomes, were excluded. Research conducted outside clinical or educational healthcare settings and publications not peer-reviewed or unavailable in English were also omitted.

### Settings

This review included studies conducted in various healthcare and educational settings where SBAR/ISBAR protocols were applied or assessed. Eligible settings included both public and private hospitals, covering primary and secondary care facilities. Studies from academic institutions—such as medical, nursing, and pharmacy schools—were considered, especially when the framework was incorporated into curricula or training simulations. Research from acute and chronic care environments, as well as medical and surgical units, was included. To ensure comprehensive coverage, studies conducted in COVID-19-affected health facilities and those in non-COVID clinical settings were both eligible.

Studies were excluded if they were conducted outside recognized healthcare or health education settings. Research from purely administrative, corporate, or non-clinical environments was deemed ineligible. Studies not relevant to patient care, clinical handovers, or professional training within the specified medical domains were excluded. Additionally, settings unrelated to hospital systems, formal care structures, or health education programs—such as community organizations without clinical components—were omitted.

### Types of sources

#### Data extraction

The Centers for Disease Control and Prevention (CDC) at McMaster University in Canada has guidelines for extracting the following information from selected articles: (1) study details, authors, title, year published, and year of research; (2) place and time; (3) study design; (4) intervention component; (5) description: what is proposed, how it is delivered, who is targeted, where it is delivered; (6) theory described; (7) setting; (8) population, sampling, and sample; (9) characteristics; (10) comparison groups; (11) primary outcomes and how and where they are assessed; and (12) secondary outcomes (22). The author (CL) evaluated all studies, and any disagreements with the second researcher (MR) were discussed between them. In case of any dispute, the opinion of a more experienced person or a third party was considered. Study quality was not a factor in the inclusion or exclusion criteria, which aligns with the Arksey and O'Malley scoping review framework (23).

#### Search strategy

**Figure 1:**
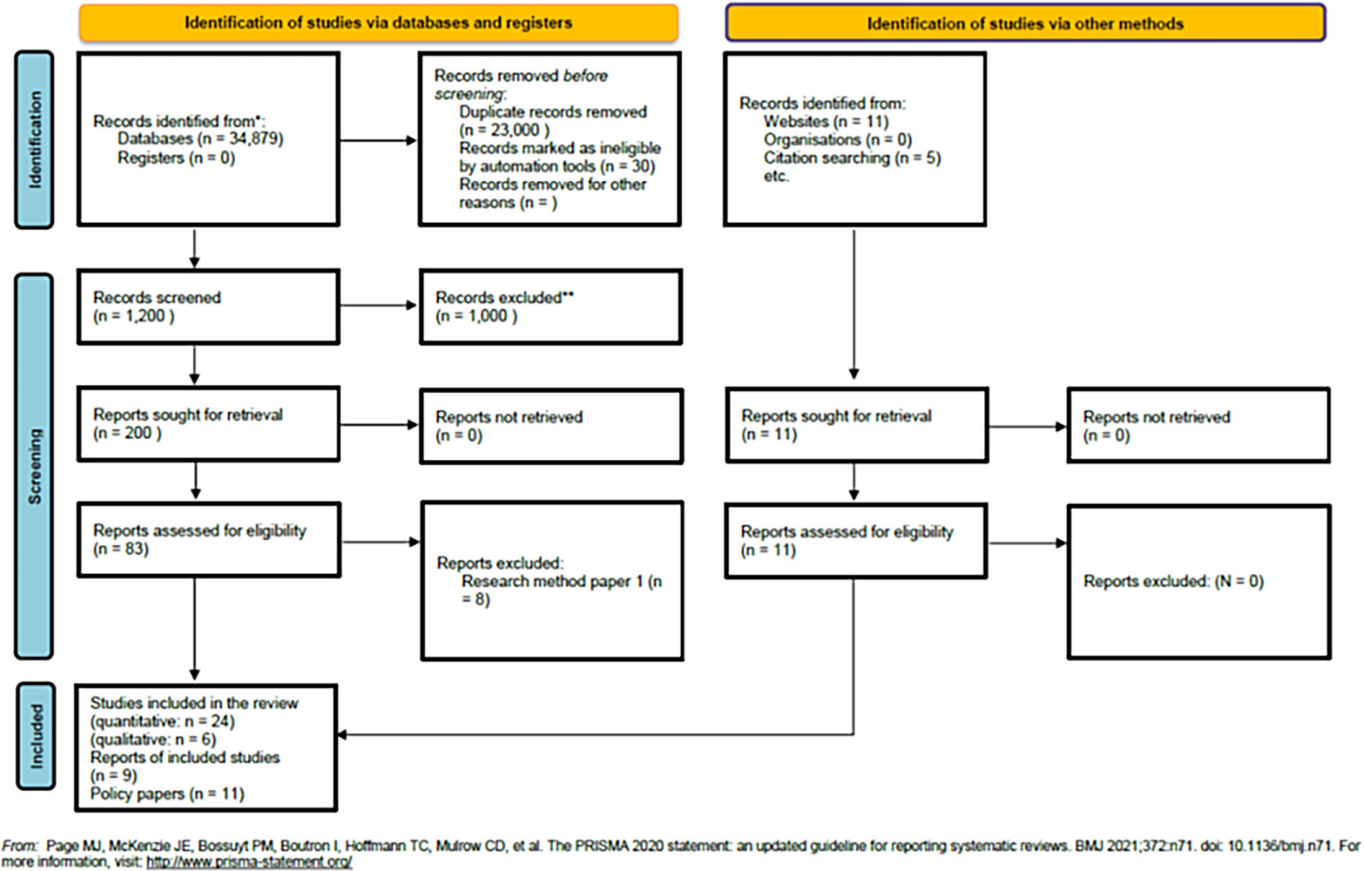
PRISMA flowchart.

In the search strategy, the core terms included “handover,” “handoff,” “SBAR,” “ISBAR,” and “COVID-19” connected by the Boolean operators “AND, OR, NOT.” During the search, thesaurus terms were organized in a tree structure, with more specific and narrower terms on the lower branches; all thesaurus terms were searched and “exploded” when we used MEDLINE Search (24). To implement the multidatabase search, the review had a focused query, a detailed description of the selected articles, a definition of central ideas, and documentation of the search process with friendly syntaxes and Boolean search (24). The primary search databases were (1) health sciences and medicine (PubMed/MEDLINE, Embase, Web of Science); (2) nursing (CINAHL); (3) psychology (PsycINFO); and (4) education (ERIC) (25). Additional sources included Google Scholar and Google. The PRISMA flowchart presented in Figure 1 summarizes the results.

#### Basic keywords

“ISBAR handover”; “clinical communication”; “structured handover”; “COVID-19 pandemic”; “non-COVID settings”; “healthcare handover”; “systematic review”; “meta-analysis”; “patient safety”; “interprofessional communication”

#### Grouped keywords with Boolean

(“ISBAR” OR “SBAR” OR “structured handover” OR “communication protocol”) AND

(“COVID-19” OR “coronavirus pandemic” OR “non-COVID” OR “general wards”) AND

(“patient safety” OR “communication quality” OR “clinical outcomes”) AND (“systematic review” OR “meta-analysis” OR “literature review”)

#### Truncated keywords

The search terms were developed to balance specificity with broad retrieval across major databases. Keywords related to the ISBAR framework included terms such as ISBAR, SBAR, handover*, hand-off*, communicat*, transfer*, shift chang*, and clinical handover. These were used to capture variations in terminology and spelling across international literature. Contextual terms addressed the clinical setting, including both COVID-19 and non-COVID environments. Relevant truncations and phrases were COVID-19, coronavirus, SARS-CoV-2, pandemic*, non-COVID, routine care, acute care, chronic care, emergenc*, hospital*, ward*, unit*, and setting. These allowed the retrieval of studies across diverse health system structures and care models. Population-based keywords targeted healthcare professionals, using nurse*, physician*, doctor*, clinician*, healthcare professional*, HCP*, student*, and trainee. This ensured the inclusion of studies involving varied clinical roles and levels of expertise. Outcome-related keywords included terms such as outcome*, effect*, impact*, efficac*, safety, error*, adverse event*, patient care, patient safety, interprofessional, collaborat*, team*, self-efficac*, and confidenc*, enabling capture of articles reporting both clinical and professional development measures. Finally, methodological terms focused on review and synthesis approaches: systematic review, meta-analys*, scoping review, integrative review, literature review, and evidence synthesis. These were critical for identifying eligible sources aligned with the study's design.

#### Combined search keywords with truncation

The *search keywords combined* yielded: (ISBAR OR SBAR OR handover* OR hand-off* OR communicat* OR transfer* OR “clinical handover”) AND (COVID-19 OR coronavirus OR SARS-CoV-2 OR pandemic* OR non-COVID OR “routine care” OR “acute care” OR “chronic care” OR emergenc* OR hospital* OR ward* OR unit*) AND (nurse* OR physician* OR doctor* OR clinician* OR “healthcare professional*” OR HCP* OR student* OR trainee*) AND (outcome* OR effect* OR impact* OR efficac* OR safety OR error* OR “adverse event*” OR “patient care” OR “patient safety” OR interprofessional OR collaborat* OR team* OR self-efficac* OR confidenc*) AND (“systematic review” OR meta-analys* OR “scoping review” OR “integrative review” OR “literature review” OR “evidence synthesis”).

#### PRISMA flowchart

During the identification stage, we extracted 34,979 records from the databases. Afterwards, 23,000 records were excluded due to duplication, while 30 records were marked as ineligible by automation tools. We found 11 records from websites and retrieved 5 additional records through citation searching. During the screening process, we reviewed 1,200 records; 1,000 were excluded based on the titles and abstracts. This resulted in 200 records being retrieved from databases and 11 others identified through alternative methods, such as hand searching or selecting from references of the studied articles. At the end of the screening, 58 records were initially deemed eligible, and 8 were excluded because they did not meet the inclusion criteria. The final sample included 24 quantitative studies, 6 qualitative studies, 9 reports, and 11 policy papers (Figure 1).

#### Statistical analysis

We converted the numerical results from each study (such as *t*-test, ANOVA *F*, correlation coefficients, and percentages) to extract their effect sizes (ES) and 95% confidence intervals (CIs) from the outcomes. They mainly used Cohen's *d*, which estimates the magnitude of an intervention (ISBAR) on the relative outcomes on a scale from 0 (nil) to 1 (huge) and transforms it into a *common language effect size* (CLES) (26). For calculating Cohen's *d* and CLES, we used the Psychometrica online calculator (https://www.psychometrica.de/effect_size.html) (27). The CLES is typically expressed as a percentage that indicates the effect of the independent variable on the dependent variable (28). The CLES indicates the likelihood (ratio) that a score randomly chosen from the intervention group would differ from a score randomly chosen from the control group when testing an intervention's effectiveness (29). Given the importance of sharing research findings in applied psychology, it is crucial to identify the most effective way to communicate ESs to general audiences is crucial (30).

The final step of the analysis was to find the 95% CIs of the CLES using an online calculator (31). Although CLES' simplicity of interpretation is its main advantage over other impact measures, it also has other appealing qualities; for example, it can be used regardless of the number of groups being compared, the degree of relatedness between the groups' scores, or the accuracy of the scores (29). Then, we calculated the fit of the CLES results using the Chi-square goodness of fit, Kolmogorov–Smirnov, and Shapiro–Wilk tests for normality. The CLES values and their 95% CI were the numerical values included in the meta-analysis. Alpha's rejection level for statistical significance in all tests was *p* ≤ 0.05, while the null hypothesis (Ho) was that the observed data distribution of data did not differ from a normally distributed one.

A meta-analysis of the subpopulation computed the global effect size (ES) and the coefficient of heterogeneity (*I*^2^) for the selected outcomes. We used the OpenMeta-Analyst of Brown University to perform a multistrata meta-analysis (32). Since the extracted data came from diverse populations and studies, we anticipated an asymmetrical funnel plot distribution explaining variations among the studies that could result in potential selection bias. Cochrane RoB-2 assessed the risk of bias for the studies included in the meta-analysis (https://methods.cochrane.org/bias/resources/rob-2-revised-cochrane-risk-bias-tool-randomized-trials). The outcomes of SBAR/ISBAR implementation included effects on communication quality, team performance, and care quality. The outcome data were continuous, dichotomous, ordinal event history, and counts (33). Moreover, further reviews resulted in increasing the number of studies deemed suitable for inclusion in this systematic review (SR) (34).

#### Ethical considerations

The current SR did not require local and university ethical approval because it is a literature review with no intervention. However, the authors are aware of the ethical considerations of SRs. They referenced the general ethical guidelines for SRs, recognizing that (1) an SR may include studies with ethical inconsistencies and diversity; (2) an SR may report unethical studies; (3) in theory, a meta-analysis of extracted data might serve a different purpose than some of the original research it includes; and (4) there is an inherent subjective element in the process of conducting an SR and meta-analysis that can produce outcomes not originally intended in the screened research screened (35). Using SRs of biased research to guide policy decisions under the assumption that review findings represent the whole population raises serious ethical issues. Therefore, systematic reviewers must carefully assess the impact of potential publishing and search biases when developing an appropriate sample and search strategy (36).

#### Data summary

The present systematic review summarized the results from the extracted articles using quantitative meta-analysis and a narrative review of qualitative outcomes. The PICOS framework was used to summarize the studies (37). The qualitative outcomes were combined based on a Setting, Perspective, Intervention, Comparison, and Evaluation (SPICE) framework that allowed for clear recommendations at the end of the scoping review; using these findings in policies, program development, and research is a method of synthesizing bibliographic searches (38). The SPICE framework aided the summary by including questions related to the (1) setting, where? (2) perspective, for whom? (3) intervention, what? (4) comparison, compared with what? and (5) evaluation, with what outcomes? (39). One step in a meta-synthesis is to compare studies and produce a final summary of the results (39). Incorporating these findings into policy, program development, and research completed the synthesis of the literature search (40).

Meta-synthesis is a qualitative interpretive method used in systematic reviews to combine findings from multiple studies that employ different methodologies, especially those of a qualitative or mixed-methods approach. Unlike meta-analysis, which statistically combines numerical data, meta-synthesis aims to identify overall patterns, themes, and theoretical insights from detailed textual data such as participant stories, thematic codes, or interpretive frameworks. This process involves systematically selecting and evaluating relevant literature, extracting findings that represent shared or different perspectives, and integrating these insights into a cohesive conceptual model that goes beyond individual study contexts (41).

Meta-synthesis is especially suited to examining phenomena that are context-dependent, nuanced, and emotionally or relationally complex—such as the experience of implementing communication frameworks such as ISBAR in healthcare environments. Through interpretive layering and thematic integration, meta-synthesis enables reviewers to identify convergence in experiential knowledge, articulate latent meanings across professional narratives, and generate evidence-informed theories that contribute to best practice in clinical communication, interprofessional collaboration, and patient-centered care. Its value lies in the ability to generate novel understandings that enrich policy development, educational interventions, and compassionate clinical practice (42).

#### Relevance

Before any critical assessment, the selected publications were examined to determine their relevance; therefore, a complete version of each study was evaluated using the inclusion and exclusion criteria (43). We followed *Saracevic's Manifestations of Relevance* for the selected studies, which can be described as follows: (1) system/algorithmic relevance, how well a query resembles the retrieved document, its adequacy, usefulness, and value in use; (2) topical relevance, the semantic match between the query and the retrieved articles, including thematic richness, breadth, value, and approach; (3) cognitive relevance, the novelty, usefulness, reliability, verifiability, and quality of information; (4) motivational relevance, how well a document aligns with a user's objectives, goals, and reasons for seeking information, including personal trust and confidence in the information; and (5) situational relevance, how the retrieved articles help the reviewer achieve the review's goal (44, 45).

#### Summary of findings

To summarize the findings, we used the summary of findings GRADEpro GDT Cochrane table, which allows the extraction of evidence certainty, effect sizes (ES), and their confidence intervals (CIs) (46). GRADEpro GDT assessment enables the analysis of bias risks in search results and studies, extracts outcomes, and determines the grade of certainty in the evidence. The domains that can be extracted using GRADEpro include (1) risk of bias at both the study and outcome levels; (2) inconsistency, with a focus on clinical heterogeneity; (3) indirectness, assessed through direct comparisons between treatments in populations and settings similar to those for which the recommendations are intended; (4) imprecision, related to the accuracy of the population estimate based on the sample; and (5) publication bias, which considers the likelihood that results have influenced published outcomes (47–49). We also conducted a narrative review of the outcomes. A narrative review enables researchers to describe what is currently known about a topic, while also providing a subjective analysis and critique of the existing literature (50). The goal was to create a meaningful synthesis of research that involves a detailed description and interpretation (50).

#### Heterogeneity

The authors assessed the statistical heterogeneity of outcomes (CLES) with the following (51, 52): (1) visual inspection of the (forest) plots of confidence intervals of CLES; (2) *I*^2^ coefficient of heterogeneity (from 0% to 100%); the Cochrane's *Q* and *χ*^2^ statistics were used to detect statistical heterogeneity, where *p* < 0.05 indicates a high level of statistical heterogeneity between studies; *I*^2^ statistic is employed to quantify the statistical heterogeneity between studies, where *I*^2^ of 30%–50% represents moderate and *I*^2^ of 50%–90% characterizes substantial heterogeneity (53). A random-effects meta-analysis was used, as preliminary results from our pilot study showed high heterogeneity in the direction of the outcomes. We also calculated the 95% CI for all the CLES scores (54). Statistical software for meta-analysis was OpenMeta-Analyst by Brown University to complete a multilevel analysis (http://www.cebm.brown.edu/openmeta/). Risk of bias was calculated using Cochrane RoB-2 software (https://methods.cochrane.org/bias/resources/rob-2-revised-cochrane-risk-bias-tool-randomized-trials).

## Results

**Figure 2:**
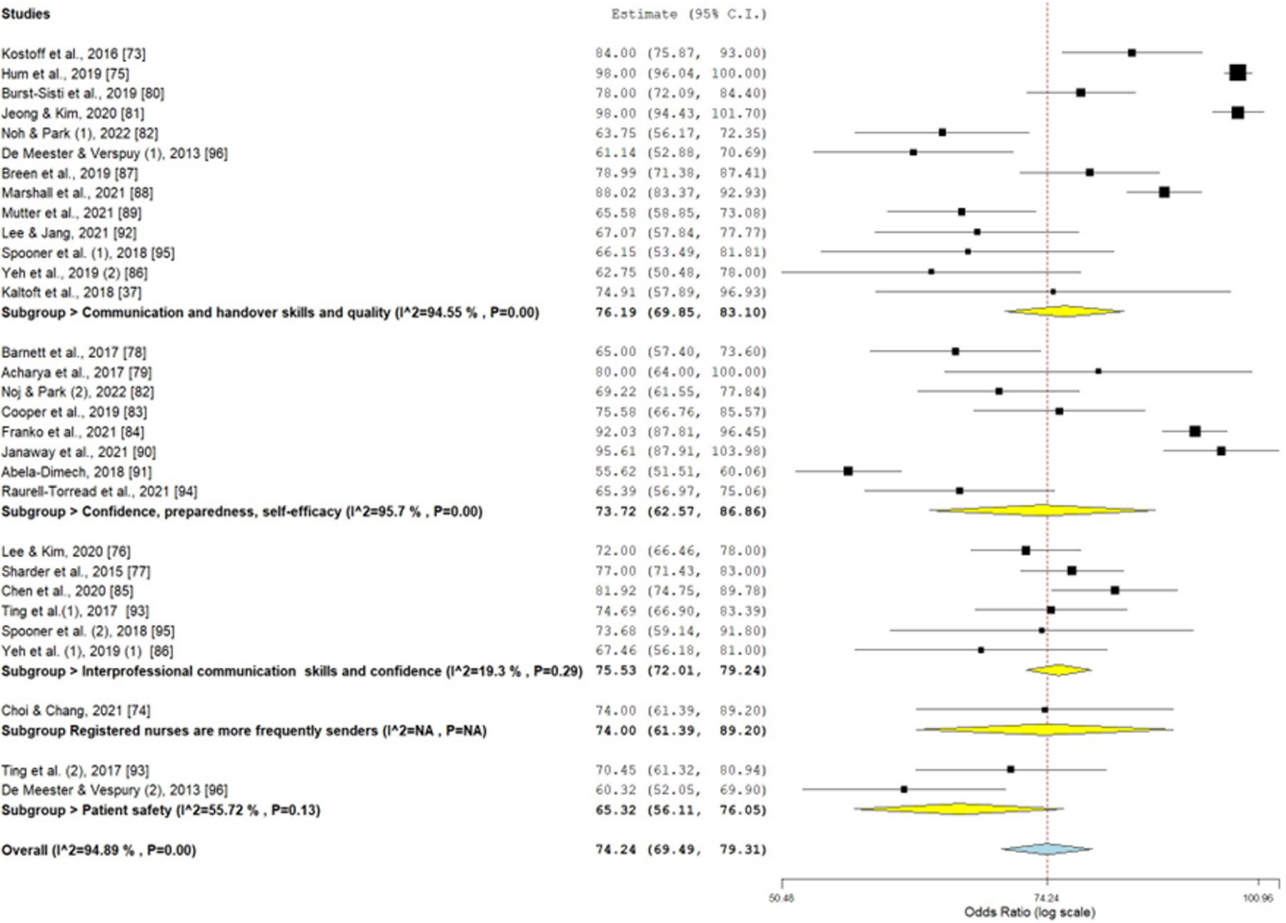
Forest plot.

**Figure 3:**
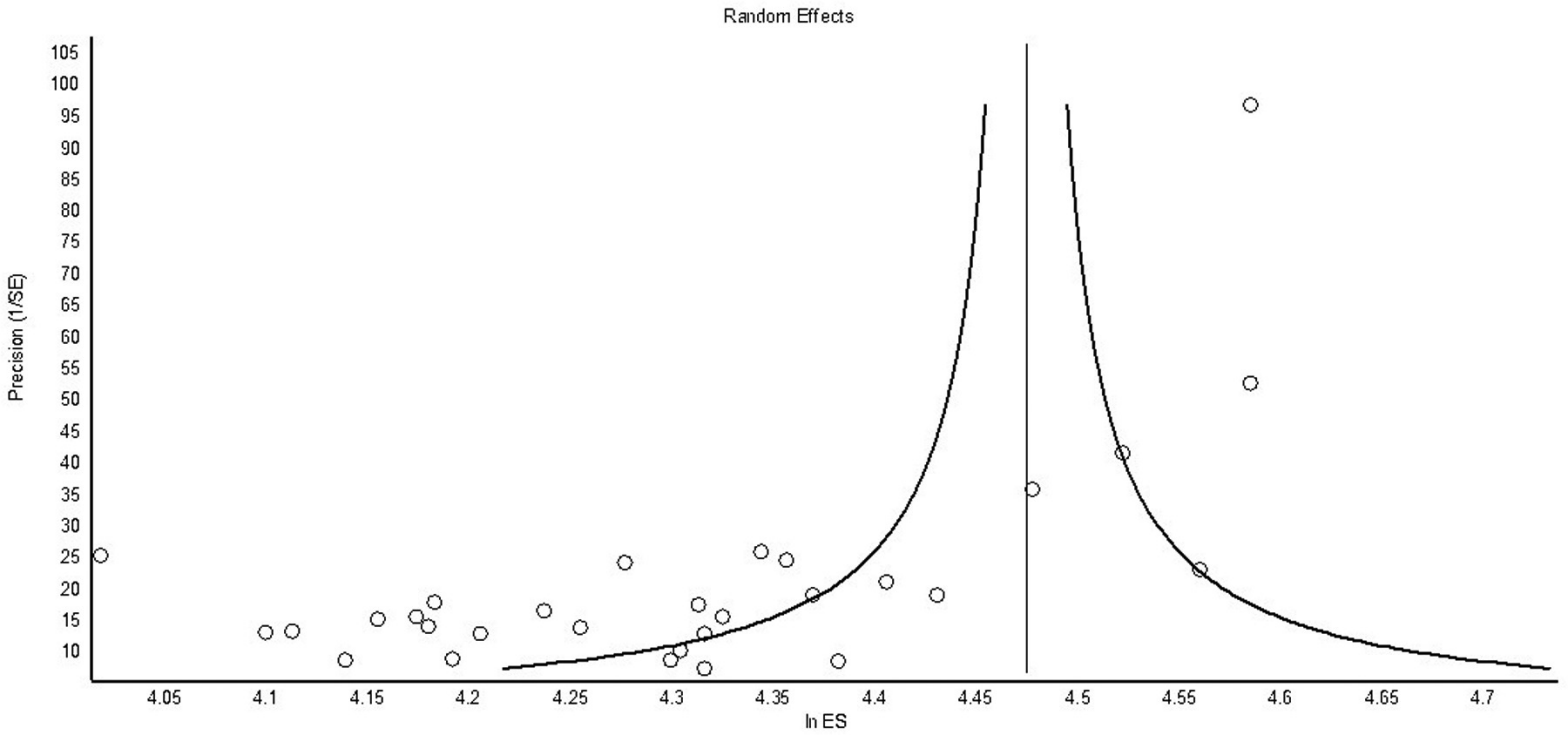
Funnel plot.

We extracted 29 studies or outcomes for the preliminary meta-analysis. The review began in January 2023 and ended in July 2025. The selected studies employed various implementation strategies and analyzed ISBAR/SBAR. Therefore, we found significant variability in the global extracted CLES (74.24%; 95% CI = 69.49%–79.31%), with *I*^2^ = 94.89% (*p* < 0.001), *τ*^2^ = 0.30, and Cochrane's *Q* = 567.224 (df = 29). The major subpopulations clustered around the following subpopulation outcomes of SBAR/ISBAR, with the extracted degree of certainty of the results as generated by the GRADEpro table and software (Figures 2, 3):

• *Increased communication and handover skills and quality* (13 studies), with CLES = 76.19% (95% CI = 69.85%–83.10%) and high heterogeneity in the outcomes (*I*^2^ = 94.55%); in GRADEpro, this outcome had a moderate certainty in the results, with critical importance for the study.
• *Increased confidence, preparedness, and self-efficacy in handover communication* (eight studies), with CLES = 73.72% (95% CI = 62.57%–86.86%) and high heterogeneity (*I*^2^ = 95.7%); in GRADEpro, this outcome had a moderate certainty, with an important impact on the study.
• *Increased interprofessional communication skills and confidence* (six studies), with CLES = 75.53% (95% CI = 72.01%–79.24%) and minor heterogeneity (*I*^2^ = 19.3%); in GRADEpro, this outcome had a moderate certainty and was critical for the study, with a low heterogeneity of the results.
• *Registered nurses were more frequently senders than receivers in interprofessional communication* (one study), with CLES = 74.00% (95% CI = 61.39%–89.20%); in GRADEpro, this study had a very low certainty and had an unimportant impact on the results.
• *Increased patient safety* (two studies) with CLES = 65.32% (95% CI = 56.11%–76.05%) with high heterogeneity (*I*^2^ = 94.14%); in GRADEpro, this reached a very low certainty, although it was important for the study.

The Kolmogorov–Smirnov test of CLES normality was *D* = 0.10 and accepted the normal distribution of the CLES (*p* > 0.10). The overall bias for all studies was low, although very few were randomized controlled studies (Tables 1–3; Figures 2, 3).

**Table 1:** Summary of findings (PICO).

Authors	Country	Setting	Study design	Sample size	Population	Intervention	Comparison and measurements	Outcome	Cohen's d	Magnitude ES	CLES (low) (%)	CLES (%)	CLES high (%)
Kostoff et al. (55)	USA	School of Pharmacy	BAS^a^	58	Pharmacy students (PharmD):	Simulation based on SBAR where pharmacy students and nursing students were, respectively, sender and receiver	Collaborative Competency Attainment Survey (ICCAS)	↑communication skills	1.39	⊕⊕⊕^b^	74.0	84.0	93.4
Choi and Chang (56)	South Korea	Nursing home emergency	Survey, mixed-method	50	Nursing home personnel	Interviews on SBAR application and network analysis on data from semi-structured, face-to-face interviews	Comparing information flow between care worker, occupational therapist, physical therapist, registered nurse, and social worker	Registered nurses are more frequent senders	0.5	⊕⊕⊕	58.8	74.0	89.2
Hum et al. (57)	South Korea	Nursing school	BAS	83	Pediatric nurses	Impact of an SBAR communication program on communication performance, perception, and practicum-related outcomes in senior-year nursing students	Intervention vs. control group	↑communication clarity and handover confidence	3.02	⊕⊕⊕	95.0	98.0	100
Lee and Kim (58)	South Korea	Nursing school	BAS, prospective observational	194	Nursing students	Team task performance was categorized into two phases: the initial team performance before a call to a mock doctor and the team task performance after receiving verbal instructions from a doctor via phone	Team performance correlation with SBAR and read-back communication in a simulated emergency	↑team performance	0.81	⊕⊕⊕	65.0	72.0	78.0
Shrader et al. (59)	USA	PharmD Fourth year	BAS	171	Pharmacy students	Simulation with standardized colleagues	Within sample; inpatient and outpatient settings	↑interprofessional communication skills and confidence	1.06	⊕⊕⊕	70.0	77.0	83.0
Barnett et al. (60)	USA	PharmD students	BAS	118	Pharmacy students	Simulated interactions with healthcare providers	Before–after SBAR preparedness to medication recommendation using the SBAR tool	↑preparedness	0.55	⊕⊕^c^	56.4	65.0	73.6
Acharya et al. (61)	USA	School of Psychiatry	BAS	11	Trainees in psychiatry	3 h sessions in simulation education	Before–after SBAR confidence in giving and receiving information	↑confidence	1.20	⊕⊕⊕	53.1	80	100
Brust-Sisti et al. (62)	USA	Pharmacy school	BAS	197	Third-year pharmacy students	Simulated telephone intervention	Before–after SBAR confidence in delivering pharmacotherapy-related intervention	↑communication skills	1.12	⊕⊕⊕	71.5	78.0	84.4
Jeong and Kim (63)	South Korea	Nursing college	BAS	54	Third-semester nursing students	Handover skills	Clarity in handover	↑clarity	3.01	⊕⊕⊕	92.7	98	101.7
Noh and Park (64)	South Korea	Nursing college	BAS	120	Fourth-year students	Simulation with 200 min sessions for 15 sessions	Comparison in communication flow timing	↑communication flow ↑self-efficacy	0.46 0.71	⊕^d^ ⊕⊕	55.15 60.96	63.75 69.22	72.35 77.84
Cooper et al. (65)	USA	Doctor of Nursing Practice programs (DNP) and Doctor of Physical Therapy (DPT)	BAS	71	First-year DPT and second-year DNP students	Educational intervention on SBAR and IPE using an online audio-conferencing tool	Comparison in the levels of communication confidence	↑confidence	0.98	⊕⊕⊕	65.59	75.58	85.57
Franko et al. (66)	USA	Nursing and medical school	BAS	144	Pharmacy, medicine, and nursing students	Education in the use of SBAR	Comparison in the use of SBAR components	↑appropriate use	1.99	⊕⊕⊕	87.61	92.03	96.45
Chen et al. (67)	China	First Affiliated Hospital of Xi’an Jiaotong University	BAS	92	General practitioners	Impact of the ISBAR communication training on residents’ interpersonal communication and teamwork in general practice standardized training	Doctor-to-nurse communication ability	↑interpersonal communication ability, doctor-to nurse ↑interpersonal communication, doctor-to-doctor	1.29 0.97	⊕⊕⊕ ⊕⊕⊕	74.06 67.5	81.92 75.36	89.78 84.17
Yeh et al. (68)	USA	Nursing school	BAS	46	Pre-licensure nursing students	Communication performance; voluntary participants must complete two deliberate practice sessions over 2 weeks with a small incentive	Comparison of participant evaluation on components of the online deliberate practice session	↑performamce ↑confidence	0.46 0.64	⊕ ⊕⊕	62.75 67.46	0.46 0.53	0.78 0.81
Breen et al. (19)	Ireland	Nursing and medical school	RCT^d^ + BAS	90	Third-year nursing students and final-year medical undergraduates	Proficiency-based progression (PBP) training approach to clinical communication in the context of a clinically deteriorating patients	PBP vs. e-learning alone or in combination with standard simulation	↑communication skills	1.14	⊕⊕⊕	70.57	78.99	87.41
Marshall et al. (69)	Australia	Medical school	RCT + BAS	168	Medical students	Communication during telephone referral in a simulated clinical setting	Clarity of delivery on a five-point Likert scale	↑clarity	5.06	⊕⊕⊕	83.11	88.02	92.93
Mutter et al. (70)	USA	Schools of Medicine and Nursing	BAS	154	Fourth-year medical students and master's nursing students	Mock-paging interprofessional education	Immediate vs. delayed feedback	↑SBAR scores in the immediate feedback group	0.56	⊕⊕	58.08	65.58	73.08
Janaway et al. (71)	UK	Mental health settings	Survey	23	Psychiatric nurses and doctors	Survey about awareness of SBAR through its use and benefits	Objective data were collected, looking at handover gathered during the survey period. Data were collected via phone from the duty physician over a 5-day period, twice daily	↑self-efficacy ↑understanding of patients	2.58	⊕⊕⊕	87.24	95.61	103.98
Abela-Dimec (72)	Canada	Mental health settings	BAS survey	481	Psychiatric nurses	Impact of SBAR on 122 handovers	Comparing the degree of satisfaction in the information received during handovers	↑satisfaction	0.20	⊕	51.18	55.62	60.06
Lee and Jang (73)	South Korea	Mental health settings	BAS	74	Psychiatric nurses	SBAR simulation to improve communication skills	SBAR vs. communication as usual	↑communication skills	0.47	⊕	56.99	67.07	77.77
Ting et al. (74)	Taiwan	Obstetric wards	BAS	96	Obstetric nurses	1 h session during monthly meetings	Nurses’ scores on the Chinese version of the Safety Attitudes Questionnaire (SAQ)	↑teamwork climate/communication ↑safety climate,	0.94 0.76	⊕⊕⊕ ⊕⊕	65.99 61.32	74.69 70.45	83.39 79.58
Raurell-Torredà et al. (75)	Spain	Nursing school	BAS RCT	93	Undergraduate nurse students	The intervention group was trained in teamwork skills, role and task assignment skills, and the use of the SBAR worksheet in a 1 h role-play training session	Intervention + control group measured on the KidSIM Team Performance Scale (teamwork skills) and the Clinical Simulation Evaluation Tool (non-technical skills)	↑confidence	0.56	⊕⊕	55.72	65.39	75.06
Spooner et al. (76)	Australia	Public hospital	BAS	35	Junior medical officers (JMO)	Following ISBAR, there is an increase in core categories that are completed	Measuring subjective responses by survey and completeness of ISBAR items	↑confidence and skills ↑quality of handovers	0.89 0.58	⊕⊕⊕ ⊕	55.5 50.5	73.68 66.15	91.8 81.8
Kaltoft et al. (39)	Denmark	Public hospital	BAS survey	50 observations	Both certified registered nurse anesthetists and registered nurses (RNs) from postanesthesia care unit	Nurses were interviewed about their satisfaction with the handover via an electronic survey	Comparing satisfaction in the handover before and after exposure to SBAR	↑quality of handovers	0.95	⊕⊕⊕	62.89	74.91	86.93
De Meester and Vespury (77)	Belgium	Public hospital	BAS	425	Nurses were taught to interact with doctors in 16 hospital wards using SBAR when patients were worsening	SBAR training of intensive care unit (ICU) nurses	Comparing perception of teamwork and efficient communication; comparing ICU admissions; comparing patient fatalities	↑communication skills and quality ↓ expected deaths	0.40 0.37	⊕ ⊕	61.14 60.32	52.88 50.60	70.69 69.90

^a^ BAS, before–after study.

^b^ Large ES.

^c^ Intermediate ES.

^d^ Small ES.

**Table 2:** GRADEpro GDT summary of findings.

Certainty assessment							No of patients	Effect size CLES	Certainty	Importance
No. of studies	Study design	Risk of bias	Inconsistency	Indirectness	Imprecision	Other considerations	SBAR handover + routine handover	(95% CI)		
Increased communication and handover skills (assessed with: I/SBAR)										
13	Observational studies	Serious	Serious	Not serious	Not serious	Publication bias strongly suspected Strong association All plausible residual confounding would reduce the demonstrated effect	14,433	76.19 (69.85–83.10)	⊕⊕⊕◯ Moderate	Critical
Registered nurses are more frequently senders (assessed with: I/SBAR)										
1	Observational studies	Serious	Serious	Not serious	Very serious	Publication bias strongly suspected Strong association All plausible residual confounding would suggest spurious effect, while no effect was observed	50	74.00 (61.39–89.20)	⊕◯◯◯ Very low	Not important
Increased interprofessional communication skills and confidence (assessed with: I/SBAR)										
6	Observational studies	Not serious	Not serious	Not serious	Not serious	Publication bias strongly suspected Strong association All plausible residual confounding would reduce the demonstrated effect	500	75.53 (72.01–79.24)	⊕⊕⊕◯ Moderate	Critical
Increased confidence, preparedness, and self-efficacy (assessed with: I/SBAR)										
8	Observational studies	Not serious	Not serious	Not serious	Not serious	Publication bias strongly suspected Strong association All plausible residual confounding would reduce the demonstrated effect	1,104	73.72 (62.57–86.86)	⊕⊕⊕◯ Moderate	Important
Patient safety (assessed with: I/SBAR)										
2	Observational studies	Not serious	Not serious	Not serious	Not serious	Publication bias strongly suspected Strong association All plausible residual confounding would reduce the demonstrated effect	518	65.32 (51.11–76.05)	⊕◯◯◯ Very low	Important

**Question:** What are the I/SBAR handover outcomes compared with routine handover for communication in healthcare?

**Setting:** Schools of medicine and nursing; pharmacy schools; psychiatric settings; Doctor of Pharmacy school; Doctor of Physical therapy school; nursing home.

**Bibliography:** Kostoff et al. (78), Choi and Chang (79), Hum et al. (80), Lee and Kim (81), Shrader et al. (82), Barnett et al. (83), Acharya et al. (84), Brust-Sisti et al. (85), Jeong and Kim (86), Noh and Park (16), Cooper et al. (87), Franko et al. (88), Chen et al. (89), Yeh et al. (90), Breen et al. (91), Marshall et al. (92), Mutter et al. (93), Janaway et al. (55), Abela-Dimec (56), Lee and Jang (57), Ting et al. (58), Raurell-Torredà et al. (59), Spooner et al. (60), Kaltoft et al. (28), De Meester and Vespury (61). [Table adapted from Schünemann et al. (47)].

**Table 3:** Summary of findings, outcomes, and RoB-2 quality assessment.

Authors	RoB-2 quality assessment^b^ Low risk: 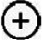 Some concerns: 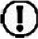 High risk: 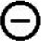						
	Outcomes^a^	D1	D2	D3	D4	D5	OA
Kostoff et al. (55)	O1	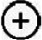	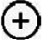	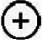	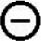	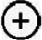	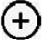
Choi nd Chang (56)	O4	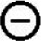	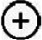	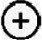	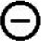	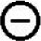	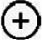
Hum et al. (57)	O1	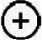	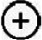	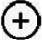	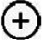	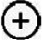	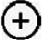
Lee and Kim (58)	O3	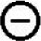	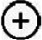	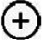	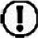	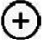	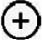
Shrader et al. (59)	O3	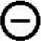	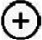	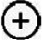	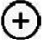	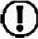	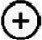
Barnett et al. (60)	O2	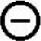	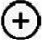	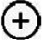	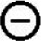	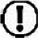	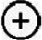
Acharya et al. (61)	O2	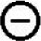	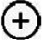	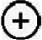	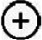	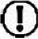	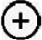
Brust-Sisti et al. (62)	O1	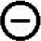	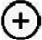	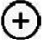	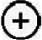	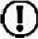	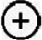
Jeong and Kim (63)	O1	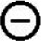	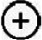	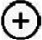	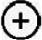	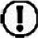	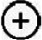
Noh and Park (64)	O1, O2	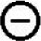	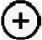	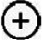	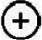	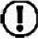	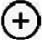
Cooper et al. (65)	O2	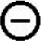	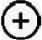	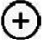	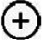	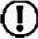	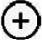
Franko et al. (66)	O2	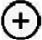	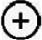	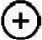	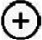	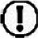	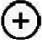
Chen et al. (67)	O3	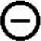	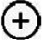	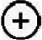	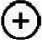	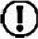	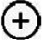
Yeh et al. (68)	O1, O2	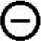	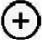	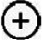	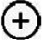	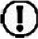	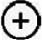
Breen et al. (19)	O1	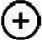	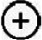	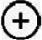	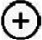	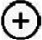	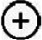
Marshall et al. (69)	O1	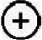	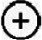	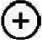	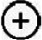	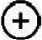	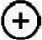
Mutter et al. (70)	O1	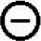	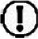	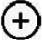	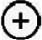	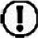	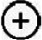
Janaway et al. (71)	O2	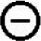	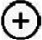	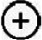	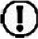	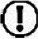	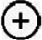
Abela-Dimech (72)	O2	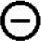	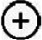	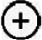	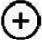	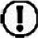	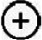
Lee and Jang (73)	O1	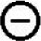	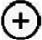	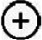	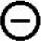	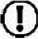	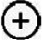
Ting et al. (74)	O2, O5	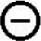	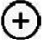	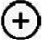	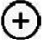	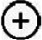	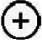
Raurell-Torredà et al. (75)	O2	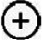	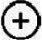	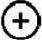	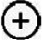	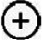	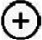
Spooner et al. (76)	O1, O3	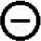	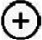	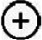	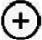	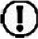	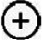
Kaltoft et al. (39)	O1	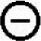	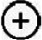	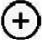	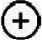	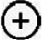	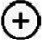
De Meester and Vespury (77)	O1, O5	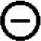	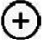	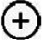	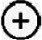	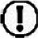	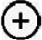

^a^ O1, increased communication and handover skills and quality; O2, increased confidence, preparedness, and self-efficacy; O3, increased interprofessional communication skills and confidence; O4, registered nurses are more frequent senders in communication exchanges; O5, increased patient safety.

^b^ D1, randomization process; D2, deviations from the intended interventions; D3, missing outcome data; D4, measurement of the outcome; D5, selection of the reported results; OA, overall.

## Qualitative critical analysis

### Handover during COVID-19

ISBAR was used during the COVID-19 pandemic to extract patient identification, analyze current scenarios, summarize background information, convey assessment, and provide recommendations; it also included identification, diagnosis, admission information, symptoms, treatments, medical background, past exposure, allergies, assessment, and nursing consensus (94). Perioperative nurses use ISBAR to summarize critical data for quick, structured handovers in COVID-19 patients, including patient names, operations, anesthesia, airway status, heat and moisture exchanger (HME) filter usage, and intraoperative issues (95). We utilized an ISBAR format for liaison psychiatric teams during COVID-19 emergencies in mental health settings; ISBAR involved recording mental symptoms and hallucinations to confirm or exclude a delirium diagnosis. The recommendation focused on psychopharmacological input—such as antipsychotics, benzodiazepines, or antihistamines—to reduce recurrent agitation in organic and agitated COVID-19 delirium (96, 97). CHs can relieve the pressure on junior doctors as well as reduce their anxiety and depression (when this generated by the medical handover); therefore, including kindness in CHs led to the coinage of the term “K-ISBAR” to emphasize the importance of delivering CHs kindly, particularly during stressful clinics during the COVID-19 pandemics (98, 99).

Other research supports the value of adopting an SBAR/ISBAR handover in the treatment of COVID-19 patients who are severely unwell (100). We suggest improving interprofessional education and handover for COVID-19 patients by providing undergraduate healthcare students with mobile technology apps for continuous ecological momentary assessment, online surveys, and mentoring (101). To address the general healthcare needs of COVID-19 patients who could not see their providers in person, students collaborated remotely with attending doctors in a multispecialty clinic using SBAR (102). During infectious disease outbreaks, online handovers using video conferencing software could be used in addition to traditional face-to-face handovers for intensive care unit patients to adhere to pandemic restrictions (78). Education improves handover completion rates for patients in an acute stage of COVID-19 but can increase workloads and staffing demands, thereby reducing handover quality (79).

### Previous systematic reviews

The first SR analyzed 18 studies and found communication training differences in ISBAR between nurses and physicians; however, it found simulation and standardized tools effective in improving CH skills (80). The second SR analyzed 18 studies and identified barriers and facilitators to communication in handover outcomes in interprofessional teams; understanding reciprocal roles and appreciating unique contributions facilitated CH, while a hostile hospital atmosphere hindered successful CHs (81). The third SR examined 28 studies and found that a lack of coordination and interprofessional communication were obstacles to patients' rehabilitation, thereby resulting in poor adherence and engagement with the clinical team (82). The fourth SR analyzed 16 studies and found that innovative mobile technology enhances accessibility, sharing, and handover communication (83). The fifth SR of 73 studies found that effective interprofessional learning strategies for CH include workshops, discussions, simulated patient training, and experiential learning (84). However, despite efforts to design communication programs around competency-based stages, we found that most programs lack a longitudinal perspective and effective means of appraising competency and self-perceived improvement in teamwork (85). The sixth SR analyzed 91 articles and found that the facilitators of effective communication include standardization, team integration, shared values, stability, and open culture; barriers include professional hierarchies, environmental factors, and irrelevant details (86). The seventh SR analyzed eight studies and found that CH reduces adverse events in patient care and improves patient safety when studies focused on team communication, with a reduction in adverse drug effects and communication breakdown in interprofessional teams (16). The eighth SR analyzed 34 studies on the SBAR technique and found significant improvements in communication clarity in classroom and clinical settings (87).

### Policy papers on SBAR implementation in the UK

The NHS Institute for Innovation and Improvement suggests learning outcomes in SBAR scenarios involve participants understanding how SBAR can improve communication, using a structured technique, discussing strategies, listing practical communication situations, simulating situations, and describing successful practice areas using SBAR (88). The Royal College of Nursing in the UK has sponsored two articles that suggest the link between team communication and clinical outcomes in patient care (89–91). The Royal College of Physicians in the UK emphasizes that using structured communication tools, such as SBAR handover, improves patient safety and that senior staff should promote a good handover culture (92). Further, the National Institute for Health and Care Excellence (NICE) in the UK encourages HCPs (such as doctors, nurses, advanced clinical practitioners, physiotherapists, mental health teams, and pharmacists) to work together to provide a planned handover of care (verbal, written, or electronic) to patients who have been hospitalized with a medical emergency (93).

## Discussion

The current review reveals that CHs are crucial for patient safety and quality of life in healthcare settings, but gaps in SBAR/ISBAR handover implementation are also reported. The COVID-19 pandemic underscored the importance of coordinated communication and prompt measures to mitigate the virus (103). In emergency rooms, electronic handovers were employed to decrease crowded multidisciplinary meetings, enhance patient safety, and minimize information governance violations, while also helping to maintain social distancing during the COVID-19 pandemic (103). Electronic handovers improve quality and efficiency, reduce clinical errors, shorten time in sharing crucial data, and enhance patient safety in intensive care units (104). Furthermore, distance and electronic handovers facilitate information sharing during COVID-19 isolation or routine multidisciplinary team meetings (MDT), thereby enabling integrated and rapid care for community patients (105). Teams can use the Internet and intranet for questions, opinions, and management changes during CHs which expedites the sharing of vital data about patients with professionals virtually and synchronously participating from different geographical areas linked to the same healthcare settings and patients (105).

One of the significant aspects that emerged in the international literature was the need to implement SBAR/ISBAR handover in clinical practice, medical education, and community health. Implementation research is crucial in global health, as it addresses the gaps and logistics of achieving national and international health objectives (106). Moreover, implementation research emphasizes partnerships among the community, people, implementers, researchers, and policymakers, focusing on strategies to improve equality, efficiency, scale-up, and sustainability (106). Implementing SBAR/ISBAR has thus a positive effect, with increased job satisfaction among healthcare professionals and teams (107). However, hospitals, units, and nurses use various delivery techniques for shift reports; therefore, narrative, repetitive, or irrelevant information may occasionally make communication difficult. Additionally, shift reports are more likely to include errors since nurse handovers occur during busy periods of the day when there are several distractions and time restrictions (108).

Therefore, implementation strategies should include daily safety-critical handovers, standardized training, structured patient handovers, uniform handover information and reception, participation of all parties, contribution of each team member, team endorsement, and professional exchanges (109). For example, ISBAR implementation reduced sepsis and operative time in a urological department compared with the non-ISBAR control group (110). In a study in nursing homes in the USA, the application of ISBAR improved communication and collaboration between home providers and nursing staff (111). In another study, nursing students found that the ISBAR has high usability, providing opportunities for active contribution and increased self-motivation (112). The findings from this systematic review also establish a strong basis for promoting wider adoption of the ISBAR communication protocol across healthcare settings. The consistent pattern observed in the reviewed studies highlights ISBAR's ability to enhance clarity, reduce cognitive load during high-pressure situations, and support a structured approach to information exchange in patient care. Importantly, these advantages translate into measurable improvements in clinical outcomes and patient safety. For instance, using ISBAR for handovers has been shown to decrease adverse events and omissions in information transfer, especially in emergency departments and intensive care units (113, 114).

From an educational perspective, ISBAR also promotes the development of communication skills among healthcare trainees. By offering a structured framework, it helps learners to understand clinical reasoning and focus on important information during interprofessional interactions (12). This organized communication training improves not only technical accuracy but also builds confidence and accountability, which are essential for delivering safe patient care (115). Furthermore, in increasingly complex and fragmented healthcare systems, ISBAR may act as a unifying tool to connect professional and disciplinary boundaries. The evidence reviewed indicates that when used consistently, ISBAR fosters a culture of standardization that encourages relational responsibility among practitioners—an idea that aligns with broader objectives of compassionate and ethically responsible healthcare delivery (116). ISBAR's role in fostering relational accountability is especially important in high-stakes environments such as pandemic response teams or community-based multidisciplinary care, where effective communication is directly linked to outcomes (117).

Future research should examine ISBAR's adaptability in culturally diverse and digitally connected healthcare settings, as well as its integration with emerging communication technologies. This will provide insights into how ISBAR can be used to promote inclusive patient engagement and support fair care delivery. Overall, the evidence reviewed in this study confirms that ISBAR is more than just a communication tool; it is a vital part of safe, collaborative, and compassionate clinical practice. Increasing its use across different systems and training programs offers a strategic way to improve patient care outcomes and should be a top priority for healthcare educators and policymakers. With the use of a structured ISBAR handover, patients reported that their interprofessional team effectively coordinated care and shared relevant information, helping to prevent communication gaps among team members (118). This approach also enhanced patient satisfaction with the healthcare team and reduced complaints related to the provision of missed or inconsistent information across providers (118).

## Conclusion

We have seen that SBAR/ISBAR handovers improve collaborative healthcare practices and communication exchanges, reduce patient risk, and increase staff satisfaction. They also enhance clinical practice by clarifying roles, reducing information gaps, and minimizing clinical and surgical risks. Therefore, structured CHs reduce medical errors, improve the quality of care, and enhance patient safety, while also reducing the global time allocated to understanding and resolving clinical cases. However, implementation and regular application remain a challenge due to high turnover and the need to address primary and secondary care emergencies. National and international policies promote the use of the SBAR/ISBAR for improved care quality, patient safety, and team performance. At other times, professionals from different backgrounds and seniority add to the same electronic notes linked to the same patient, thereby causing some interprofessional misunderstanding due to the technical language used that is unique to each HCP. Occasionally, CHs are not comprehensive and do not incorporate the ISBAR algorithm, thereby comprising bits of globally inapplicable information (119, 120).

## Limitations

This SR has several limitations. Although we conducted a comprehensive extraction of significant studies, the outcomes were heterogeneous, and we restricted the numerical analysis to span different outcomes. A more comprehensive and extended study selection would have yielded different results. SBAR/ISBAR is not the only technique used for handovers in clinical practice; overall, the study may have had a positive bias toward this approach and its use. The scrutinized studies also did not provide evidence that the proposed use of the clinical handovers had long-term effects, and whether, if adopted, the teams continued to perform well long after the training. Furthermore, almost all studies were cross-sectional, lacking randomization and blinding, which limits the conclusions regarding the association of variables but does not allow for further conclusions about the impact of biases in the selected studies.

## Data Availability

The original contributions presented in the study are included in the article/Supplementary Material, further inquiries can be directed to the corresponding author.
